# A Comprehensive Review of Cryptogenic Stroke and Atrial Fibrillation: Real-World Insights Into the Role of Insertable Cardiac Monitors

**DOI:** 10.7759/cureus.70369

**Published:** 2024-09-28

**Authors:** Faizan Khan, Anuj Varma, Priyanka K Negandhi, Sourya Acharya, Sunil Kumar, Vinit Deolikar

**Affiliations:** 1 Internal Medicine, Jawaharlal Nehru Medical College, Datta Meghe Institute of Higher Education and Research, Wardha, IND

**Keywords:** anticoagulation therapy, atrial fibrillation, cryptogenic stroke, insertable cardiac monitors, long-term monitoring, stroke prevention

## Abstract

Cryptogenic stroke, a subtype of ischemic stroke with no identifiable cause after comprehensive evaluation, presents a unique challenge in stroke prevention. Atrial fibrillation (AF), a common risk factor for ischemic stroke, is often underdiagnosed in these patients due to its intermittent, asymptomatic nature. Early detection of AF is critical, as anticoagulation therapy significantly reduces the risk of recurrent stroke in AF patients. However, traditional short-term monitoring methods frequently fail to identify paroxysmal AF. Insertable cardiac monitors (ICMs) offer a novel solution by providing continuous, long-term heart rhythm monitoring, which has proven effective in detecting occult AF. Real-world data further support the clinical value of ICMs in guiding the initiation of anticoagulation therapy, ultimately improving stroke prevention strategies. Despite some limitations, such as false positives and the invasive nature of the device, ICMs have emerged as a critical tool in modern stroke management. As technology evolves, future advancements may further enhance AF detection by integrating artificial intelligence and wearable devices. This review provides a comprehensive overview of the role of AF in cryptogenic stroke, the importance of early detection, and the growing significance of ICMs in clinical practice.

## Introduction and background

Cryptogenic stroke (CS), a type of ischemic stroke with no identifiable cause despite thorough diagnostic evaluation, poses a significant challenge in stroke management [1]. Accounting for up to 30% of ischemic strokes, it often leaves clinicians without a clear path for secondary stroke prevention [2]. Standard diagnostic approaches, such as brain imaging, echocardiography, and vascular assessments, frequently fail to uncover a definitive etiology, categorizing these strokes as cryptogenic [3]. This lack of clarity presents a critical concern, as preventing recurrent stroke in this population hinges on identifying hidden causes, one of which is often atrial fibrillation (AF) [4].

AF, a common cardiac arrhythmia, is a well-established risk factor for stroke. However, its paroxysmal nature, where episodes are intermittent and often asymptomatic, makes it an underdiagnosed cause of CS [5]. Silent AF, in particular, eludes detection through routine short-term monitoring methods, such as standard electrocardiography (ECG) or 24- to 48-hour Holter monitoring. Consequently, many patients with CS remain untreated for AF despite its potential role in their condition. The link between AF and CS emphasizes the need for advanced diagnostic tools to detect occult arrhythmias [6]. Early detection of AF in patients with CS is crucial for secondary stroke prevention. Anticoagulation therapy, the mainstay for preventing stroke in AF patients, significantly reduces the risk of recurrent events [7]. Without timely identification of AF, patients with CS may remain on less effective antiplatelet therapy, leaving them vulnerable to future strokes. Therefore, a prolonged and continuous cardiac monitoring strategy is essential for uncovering AF that may go undetected during short-term evaluations [8].

Insertable cardiac monitors (ICMs), also known as implantable loop recorders, have emerged as a vital tool in the quest to detect occult AF in CS patients. These small, subcutaneously implanted devices provide continuous, long-term heart rhythm monitoring, allowing for the detection of intermittent AF episodes that may otherwise go unnoticed. ICMs have demonstrated superior diagnostic yields in identifying AF in patients with CS, prompting a shift in how clinicians approach stroke diagnosis and management [9]. As a result, ICMs are gaining prominence in clinical practice, playing a critical role in enhancing stroke prevention strategies by enabling early AF detection and guiding the initiation of anticoagulation therapy [10]. The growing role of ICMs in stroke management reflects an important evolution in the care of CS patients [11].

## Review

CS: epidemiology and pathophysiology

CS poses a substantial challenge in neurology, accounting for approximately 25% to 40% of all ischemic strokes [12]. This condition is defined by the absence of a clear cause despite comprehensive diagnostic evaluation. Gaining a deeper understanding of the epidemiology, potential causes, pathophysiological mechanisms, and diagnostic difficulties surrounding CS is essential for improving patient outcomes [12]. The epidemiology of CS underscores its prevalence, making up 25% to 40% of ischemic strokes, with estimates suggesting that roughly one in three ischemic strokes falls into this category [13]. The incidence of CS varies across populations, with the African American and Hispanic populations being at higher risk compared to the Caucasian population. Furthermore, the recurrence rate of CS is notably high, with approximately 25% of stroke survivors experiencing another stroke within two years. This highlights the urgency of identifying potential underlying causes to implement effective secondary prevention strategies [14].

The pathophysiology of CS is multifaceted, with several underlying mechanisms potentially contributing to its occurrence. Undetected AF is a significant factor that can greatly increase stroke risk, with occult paroxysmal AF thought to account for a large portion of CSs [15]. Another potential cause is patent foramen ovale (PFO). This congenital heart defect allows blood to bypass the lungs, leading to paradoxical embolism when a clot crosses from the venous to the arterial system and causes a stroke. Additional contributing factors include aortic arch atheroma, where plaque buildup in the aorta can lead to embolic strokes, and hypercoagulable states that increase blood clot formation. In rare instances, infections, malignancies, or arterial dissections may also be implicated in CSs [16].

Diagnosing CS presents several challenges. The term "cryptogenic" covers a range of scenarios, including strokes with no identifiable cause after extensive testing, those with multiple potential causes, or incomplete evaluations. The variability in diagnostic approaches and the transient nature of some underlying conditions, such as AF, complicate efforts to pinpoint the stroke's etiology. Continuous cardiac monitoring is often necessary to detect intermittent AF, which can be elusive during initial assessments, further complicating the diagnostic process [17]. The conventional diagnostic workup for CS involves a comprehensive approach. This typically includes imaging studies, such as CT or MRI of the brain, to identify infarcts and rule out hemorrhagic strokes and vascular imaging (magnetic resonance angiography (MRA) and computed tomography angiography (CTA)) to assess for large-vessel disease [18]. An initial electrocardiogram (ECG) is performed to check for AF. However, longer-term monitoring (e.g., with a Holter monitor or ICMs) may be required to detect paroxysmal AF that is not evident during a standard ECG. Laboratory tests are also conducted to evaluate for hypercoagulable states, infections, and other systemic causes. Additionally, echocardiography assesses cardiac structure and function to identify PFO or other cardiac abnormalities. Despite these thorough efforts, many patients continue to be classified as having a CS, underscoring the need for ongoing research and improved diagnostic techniques to better identify underlying causes and reduce recurrence rates [19]. The relationship between AF and stroke risk is outlined in Table 1.

**Table 1:** Atrial fibrillation and its impact on stroke risk AF: atrial fibrillation, ICM: implantable cardiac monitor, ECG: electrocardiogram.

Category	Key Points	Impact on Stroke Risk	Clinical Implications
Relationship between AF and stroke [20]	AF causes irregular heartbeats, leading to poor blood flow and clot formation in the atria	Increases the risk of ischemic stroke due to potential embolization of clots to the brain	Early detection and management of AF is critical to reducing stroke risk
Paroxysmal and silent AF [21]	AF can be intermittent (paroxysmal) or asymptomatic (silent), making it harder to detect	Silent AF episodes are a significant risk factor for cryptogenic stroke due to undiagnosed and untreated AF	Continuous monitoring (e.g., ICMs) is important for detecting silent or paroxysmal AF
Undiagnosed AF and stroke risk [22]	Many patients with AF are undiagnosed, contributing to recurrent stroke risk	Undiagnosed AF leads to missed opportunities for stroke prevention strategies, increasing recurrence rates	Prolonged monitoring and increased awareness of AF in stroke patients are necessary to prevent recurrent strokes
Stroke risk assessment in AF [23]	Stroke risk in AF patients is often assessed using scoring systems like CHA_2_DS_2_-VASc	Higher scores indicate a greater risk of stroke, guiding the need for anticoagulation therapy	Regular use of validated stroke risk stratification tools in clinical practice to optimize treatment plans
Standard AF detection methods [24]	ECG, Holter monitoring, and external loop recorders are commonly used to detect AF	Short-term monitoring may miss intermittent AF episodes, leading to underdiagnosis	ICMs offer longer-term continuous monitoring, improving AF detection
Impact of AF on recurrent stroke [25]	AF significantly increases the risk of recurrent strokes in patients with a history of stroke	Early diagnosis and anticoagulation therapy can significantly reduce the risk of recurrent strokes	Emphasis on aggressive monitoring and anticoagulation management for stroke survivors with AF

AF and stroke risk

AF is a well-established independent predictor of ischemic stroke, increasing stroke risk by four to five times compared to individuals without AF. This elevated risk is primarily driven by the prothrombotic state associated with AF, which fosters thrombus formation [26]. Mechanisms such as atrial dilation, endothelial dysfunction, and the release of coagulation factors all contribute to the higher stroke risk in patients with AF. Given this strong association, healthcare providers must identify and manage AF promptly to reduce the risk of stroke [26].

Silent or asymptomatic AF is particularly prevalent among stroke patients, with neuroimaging studies estimating its occurrence to range from 11% to 48%. Paroxysmal AF, characterized by intermittent episodes that may resolve spontaneously, is a frequent cause of cryptogenic ischemic strokes or transient ischemic attacks (TIAs) [27]. Research suggests that a considerable number of patients who experience CSs may have undiagnosed AF. For example, a study involving 58 CS patients revealed that 41% were diagnosed with AF after an average monitoring period of six months using ICMs, highlighting the importance of diligent monitoring in this population [28].

The presence of silent, undiagnosed AF significantly increases the risk of recurrent ischemic strokes. However, early detection and effective management of AF can mitigate this risk. In the study above, none of the 24 patients diagnosed with AF experienced a recurrent stroke during follow-up, thanks to timely anticoagulation therapy. This emphasizes the critical role of early AF identification and treatment in preventing further strokes and improving patient outcomes [10].

Conventional methods for detecting AF, such as 24- to 72-hour Holter monitoring, have limitations, with a low detection yield of just 3% to 5% following a stroke. These traditional techniques often fail to capture paroxysmal and asymptomatic AF episodes due to their sporadic nature. In contrast, continuous monitoring with ICMs has proven more effective in identifying AF in patients with CSs. Current guidelines recommend at least 30 days of cardiac rhythm monitoring for individuals who have experienced an acute CS or TIA. Extended monitoring with ICMs captures significantly more AF cases than shorter monitoring durations, ultimately leading to improved management and reduced stroke recurrence [29].

ICMs: technology and mechanism

ICMs are sophisticated devices designed for continuous cardiac monitoring, particularly for detecting AF and other cardiac irregularities. A key feature of ICMs is their capacity for long-term monitoring, often spanning several years [30]. This extended monitoring capability is crucial for identifying intermittent AF episodes that may go undetected with traditional methods. Modern ICMs, like the Reveal LINQ™ (Medtronic, Minneapolis, Minnesota), have a battery life of approximately three years, allowing continuous monitoring without frequent device replacement. These devices also employ wireless technology for data transmission. For instance, the CareLink™ Network (Medtronic) facilitates automatic nightly uploads of patient data, enabling real-time monitoring and timely interventions when AF episodes are identified [31].

ICMs function by continuously recording electrocardiogram (ECG) signals. Using specialized algorithms, the devices analyze these signals to detect AF episodes, particularly those lasting more than two minutes. This capability allows healthcare providers to review the initial two minutes of ECG data for each detected AF episode, offering vital insights into the patient's cardiac condition. Additionally, many ICMs are equipped with accelerometers to track patient activity, providing context for AF episodes and helping healthcare providers understand patient behavior during these events [32].

ICMs offer several advantages over traditional cardiac monitoring methods like Holter monitors. One significant advantage is the duration of monitoring. While Holter monitors typically record cardiac activity for just 24 to 48 hours, ICMs can continuously monitor patients for years, significantly increasing the likelihood of capturing transient AF episodes that might otherwise be missed [33]. Furthermore, ICMs are implanted subcutaneously, enhancing patient comfort and eliminating the need for external devices. This design also promotes better adherence to monitoring protocols. Additionally, the automated data transmission feature of ICMs reduces the burden on patients and healthcare providers, allowing for more efficient analysis and timely intervention [34].

Several ICM devices have received FDA approval, with the Reveal LINQ being one of the most widely used. These devices are primarily indicated for detecting AF, especially in patients who have experienced CSs or unexplained syncope [35]. The early detection of AF can significantly influence treatment decisions, particularly in determining the need for anticoagulation therapy. Numerous studies have demonstrated the clinical effectiveness of ICMs in improving AF detection rates, which, in turn, enhances patient outcomes by enabling timely interventions and reducing the risk of recurrent strokes or complications associated with undiagnosed AF [35].

Real-world insights on the use of ICMs in CS

Clinical trials and observational studies have increasingly focused on the effectiveness of ICMs in detecting AF in patients with CS. One of the most significant studies in this area is the Cryptogenic Stroke and Underlying Atrial Fibrillation (CRYSTAL AF) trial, which demonstrated that ICMs substantially increase AF detection rates compared to standard care [36]. Continuous monitoring in this trial allowed for identifying AF episodes that may remain undiagnosed, ultimately leading to more effective management of patients with CS. In real-world settings, studies have shown that ICMs can detect AF in approximately 41% of patients within six months post-implantation, with detection rates increasing over time [36].

The duration and frequency of AF detection using ICMs are critical factors that enhance their utility. Research shows cumulative AF detection rates rise steadily with longer monitoring periods [37]. For example, detection rates at one month hover around 4.1%, increase to 12.2% at six months, and reach as high as 28.5% by 36 months. This progressive increase underscores the value of prolonged monitoring, as AF episodes can vary significantly, with some lasting over an hour and others exceeding 24 hours. These findings highlight the need for ongoing surveillance in patients with CS to capture intermittent episodes of AF [38].

When comparing real-world evidence with clinical trials, it becomes clear that while trials provide controlled settings to assess ICM efficacy, broader clinical practice presents challenges [39]. For example, real-world patient populations often exhibit greater diversity in comorbidities and demographics, which can influence AF detection rates. Additionally, practical issues such as device implantation logistics and patient adherence to follow-up monitoring can impact results. Nevertheless, ICMs have demonstrated superior AF detection rates to intermittent monitoring strategies like Holter monitors, which often have lower sensitivity and specificity for AF detection [40].

The primary advantage of prolonged AF detection through ICMs lies in their ability to identify asymptomatic AF, which is crucial for secondary stroke prevention. Continuous monitoring facilitates the timely initiation of anticoagulation therapy, significantly reducing the risk of recurrent strokes [41]. Studies have shown that patients in whom AF was detected following ICM implantation were appropriately treated with anticoagulants, and none experienced recurrent strokes during follow-up. This emphasizes the critical role of early and accurate AF detection in improving patient outcomes and highlights the potential of ICMs to become a standard practice in managing CS [10,41-44].

Clinical outcomes and benefits of ICM-based AF detection

The early detection of AF in patients with CS using ICMs has significant implications for stroke prevention and anticoagulation therapy. When AF is detected following an ischemic stroke, most patients can initiate oral anticoagulation (OAC) or receive other targeted stroke-preventive treatments [11]. Anticoagulation therapy has been consistently shown to reduce the risk of stroke in patients with AF. For example, in the CRYSTAL AF study, 97% of patients with ICM-detected AF were prescribed OAC within one year by their physicians, underscoring the strong likelihood of initiating anticoagulation when AF is identified through ICMs [36]. However, the STROKE AF trial did not show a significant reduction in recurrent stroke rates in the ICM-monitored group compared to standard monitoring. This outcome may be due to the study's limited sample size and the fact that 24% of patients with detected AF did not receive anticoagulation. Further research is needed to clarify the impact of ICM-guided anticoagulation on long-term stroke outcomes [36].

ICMs have been demonstrated to significantly improve the detection of AF in patients with CS compared to traditional monitoring methods. AF detection rates using ICMs range from 16% at 30 days to 21-25% at one to two years [45]. Identifying AF in these patients is critical, as those with ICM-detected AF are more likely to be prescribed anticoagulation therapy, potentially reducing their risk of recurrent strokes. The continuous, long-term monitoring provided by ICMs allows clinicians to detect asymptomatic or short AF episodes that go unnoticed with traditional monitoring techniques. This capability enables more informed decisions regarding anticoagulation and overall stroke prevention strategies [45].

While ICMs are generally more expensive than standard monitoring options, their potential to improve AF detection and facilitate targeted anticoagulation therapy may make them cost-effective in the long run. Further research is needed to assess the overall cost-effectiveness of ICMs in clinical practice. Additionally, ICMs may reduce healthcare utilization by minimizing the need for repeated monitoring sessions and clinic visits. Continuous monitoring through ICMs is often more efficient than intermittent monitoring, such as Holter monitoring, leading to better resource allocation in healthcare settings [44].

ICMs also have a positive effect on patient adherence and quality of life. The continuous nature of ICM monitoring eliminates the need for patients to comply with external devices, improving adherence compared to intermittent monitoring methods. Additionally, real-time monitoring and rapid detection of AF can provide patients with greater peace of mind, potentially enhancing their quality of life. However, the specific impact of ICMs on patient-reported outcomes has not been extensively studied, highlighting a key area for future research [46].

Limitations and challenges in the use of ICMs

The use of ICMs for detecting AF in patients with CS indeed comes with limitations and challenges that affect their overall effectiveness and acceptance. One of the most significant challenges with ICMs is the high rate of false positives in AF detection. Reported false positive rates vary between 48% and 96%, often due to artifacts like premature beats, T-wave oversensing, or motion artifacts. These false positives can create unnecessary anxiety for patients and lead to changes in treatment plans based on inaccurate data, complicating the decision-making process for clinicians. Despite improvements in detection algorithms, these misclassifications remain a key issue with ICM technology [42].

The invasive nature of ICM implantation also poses a barrier. Patients may be reluctant to undergo the implantation procedure due to concerns about the surgical risks or discomfort. This can result in the underutilization of ICMs, especially among those who might benefit most, such as patients with unexplained strokes. Although complications from ICM implantation are relatively rare, the perception of invasiveness contributes to some patients declining the procedure [47].

Data interpretation from ICMs requires specialized knowledge and careful adjudication to distinguish true AF episodes from artifacts. This process can be time-consuming and require expert review, which adds another layer of complexity. Furthermore, the variability in detection algorithms across different ICM devices may lead to inconsistent results, making it challenging to compare findings across studies or in clinical practice [47].

To mitigate these challenges, integrating artificial intelligence (AI) into ICM technology could be a promising solution. AI algorithms have demonstrated the potential to improve arrhythmia detection by reducing the number of false positives. Studies suggest that AI can filter out nonarrhythmic events and reduce false positives by up to 98%. This would help streamline data interpretation, allowing for more reliable and accurate results and ultimately improving clinical decision-making [48].

While ICMs offer valuable benefits for AF detection in CS patients, challenges like false positives, invasiveness, and the complexity of data interpretation need to be addressed. Integrating AI technology offers a future pathway to enhance their accuracy, reduce the burden on clinicians, and increase patient acceptance [48]. Limitations and challenges in the use of ICMs are shown in Table 2.

**Table 2:** Limitations and challenges in the use of ICMs AF: atrial fibrillation, ICM: implantable cardiac monitor, AI: artificial intelligence.

Category	Challenges/Limitations	Potential Impact	Mitigation Strategies
Technical limitations [49]	False-positives: Misinterpretation of non-AF events as AF episodes	Unnecessary follow-ups, patient anxiety, and inappropriate anticoagulation	Improved algorithm refinement and software updates to reduce misdetection
	Battery life: Limited battery duration may affect long-term monitoring	Device replacement or malfunction, leading to gaps in AF detection	Develop longer-lasting batteries and optimize device performance
Patient-related issues [50]	Invasive nature: ICMs require minor surgical procedures for insertion	Patient discomfort, potential complications (e.g., infection, scarring), lower acceptance rates	Improved device miniaturization and less invasive implantation techniques
	Patient adherence: Reluctance to use the device consistently or attend follow-ups	Incomplete monitoring data impacting diagnosis and management	Enhanced patient education, telemedicine support, and user-friendly devices
Data interpretation [51]	Interpretation of data: Challenges in distinguishing clinically significant AF from nonsignificant episodes	Over/under-treatment based on inaccurate data interpretation	Advanced AI-driven algorithms and increased clinician training to ensure accurate data interpretation
Cost and resource utilization [52]	Cost: High initial cost of device, implantation, and follow-up care	This may limit accessibility, particularly in resource-constrained settings	Health insurance coverage expansion, value-based care models, and cost-effectiveness studies
	Healthcare resource utilization: Frequent monitoring may increase the workload on clinicians	Increased healthcare provider burden and potential delays in response to critical data	Integration of automated alerts and remote monitoring platforms to streamline clinical workflows
Data security and privacy [53]	Privacy concerns: Transmission of sensitive health data through wireless means	Risks of data breaches and unauthorized access to patient information	Strengthening cybersecurity protocols and implementing stringent patient consent procedures for data sharing
Device performance [54]	Signal quality: Variability in signal quality, particularly in obese patients or those with scar tissue	Reduced accuracy in detecting AF episodes and generating reliable data	Continuous hardware advancements and customized calibration for high-risk patient groups

Future directions in CS and AF detection

Emerging technologies and advancements in personalized medicine are transforming the landscape of CS and AF detection. This evolution includes integrating wearable devices, artificial intelligence (AI), and improvements in stroke risk stratification through biomarkers and genomics [55]. Wearable devices, such as smartwatches and portable ECG monitors, are increasingly important for continuous AF monitoring. These devices allow real-time data collection and timely alerts for potential AF episodes [56]. Incorporating machine learning (ML) algorithms into these devices enhances their ability to detect AF early, even in asymptomatic patients. Research indicates that deep learning algorithms, particularly convolutional neural networks (CNNs), can accurately identify AF from ECG signals, making these devices suitable for large-scale applications [56].

AI is revolutionizing AF detection by automating ECG data analysis. Recent advances in AI algorithms have demonstrated significant improvements in detecting AF during normal sinus rhythm compared to traditional methods [57]. For example, AI models have achieved an area under the receiver operating characteristic curve (AUC) of up to 0.80 in predicting paroxysmal AF from single-lead ECGs, showing strong potential for widespread clinical use. However, challenges persist, such as deploying complex algorithms on wearable devices with limited processing power and ensuring robust signal preprocessing [57].

Advancements in biomarker research and genomics are also enhancing stroke risk stratification in patients with CS. Biomarkers like N-terminal B-type natriuretic peptide (NT-proBNP) help identify individuals at high risk for undetected AF, guiding more targeted screening efforts. Moreover, genomic studies reveal genetic predispositions to AF, potentially leading to personalized prevention strategies based on an individual’s genetic profile [58].

The concept of personalized medicine is gaining momentum in managing stroke and AF. Prevention strategies can be optimized by tailoring treatment plans according to individual risk factors, including genetic predispositions and biomarker levels. This may involve using anticoagulants for high-risk patients through advanced screening techniques, ensuring timely and effective treatment [59].

Finally, integrating data from ICMs with other diagnostic tools is crucial for comprehensive stroke care. ICMs provide valuable long-term monitoring data that can be combined with results from wearable devices and traditional ECGs. This multifaceted approach improves AF detection accuracy and supports better-informed clinical decisions regarding anticoagulant therapy and stroke prevention strategies [60]. Future directions in CS and AF detection are outlined in Table 3.

**Table 3:** Future directions in cryptogenic stroke and atrial fibrillation detection AF: atrial fibrillation, AI: artificial intelligence, ICM: implantable cardiac monitor, ECG: electrocardiogram.

Category	Technological Advancements	Potential Impact	Challenges/Considerations
Emerging technologies [56]	Wearable devices: Continuous monitoring with smartwatches and patches	Improved early detection of AF episodes in real-time	Accuracy of wearable sensors; patient adherence
	AI-based algorithms: Machine learning for pattern recognition and AF prediction	Enhanced ability to detect silent or paroxysmal AF; reduced diagnostic delay	Data integration with clinical systems; need for large datasets to train algorithms
Advancements in risk stratification [61]	Biomarkers: Identifying novel blood or genetic markers for stroke risk	More personalized risk assessment for recurrent strokes	Limited availability of validated biomarkers; cost implications
	Genomics: Personalized medicine approach based on genetic predispositions	Tailored anticoagulation and stroke prevention strategies based on individual profiles	Ethical concerns: access to genetic testing
Enhanced diagnostic modalities [61]	Combination diagnostics: Integrating ICM data with imaging, ECG, and wearable devices	Comprehensive stroke care; better identification of high-risk patients	Complexity of combining data from multiple sources; cost of multimodal diagnostics
Advances in device technology [62]	Miniaturized ICMs: Smaller, less invasive cardiac monitors with longer battery life	Increased patient comfort and longer monitoring durations	Device durability and signal quality with miniaturization
	Remote monitoring and telemedicine integration: ICMs transmitting real-time data to clinicians	Improved access to care, especially in remote areas; faster clinical decision-making	Data security and privacy concerns; infrastructure for remote monitoring
Innovative treatment approaches [63]	AI-driven treatment protocols: Using AI to recommend personalized anticoagulation therapies	Optimized treatment plans based on continuous AF data and patient characteristics	Clinical validation required; regulatory approval for AI-based medical decision-making

## Conclusions

In conclusion, CS represents a substantial clinical challenge due to its elusive nature and the difficulty in identifying underlying causes such as AF. The frequent underdiagnosis of AF, particularly its paroxysmal and asymptomatic forms, underscores the importance of advanced, prolonged monitoring tools in stroke prevention. ICMs have emerged as a transformative solution, providing continuous, long-term surveillance that significantly enhances AF detection in CS patients. By facilitating the early diagnosis of AF, ICMs enable timely initiation of anticoagulation therapy, reducing the risk of recurrent stroke and improving patient outcomes. As evidence from clinical trials and real-world applications continues to validate their effectiveness, ICMs are becoming an integral component of CS management, paving the way for more personalized and proactive approaches to stroke prevention.
